# The College of Nursing: A Call to Action

**Published:** 1916-12-02

**Authors:** 


### THE MATRONS' AND SISTERS' DEPARTMENT.

### THE COLLEGE OF NURSING.

### A Call to Action.

The College of Nursing has connected with its management a great array of nursing talent, comprising some of the best known workers and nearly all the best minds in the nursing profession. They, by their acceptance of seats on the Council, have undertaken the responsibility of supporting Mr. Stanley and seeing the College safely through its infancy and establishment. The time has nearly come, if it has not already arrived, when these ladies will have the privilege and duty to take an active part in propaganda for the College. Those of them who have the gift of public speaking might helpfully prepare themselves without delay for the campaign which lies before them in the immediate future. Mrs. Bedford Fenwick, having withdrawn herself from co-operation in the difficult work of organising the nursing profession, enlarging its outlook, increasing its democratic character, and providing the surest opportunities for education and advancement in life for the maximum number of British nurses, has left the field open and free to its cultivation by the able women who constitute a large proportion of the Council of the College of Nursing. It is women's work to bring the nurses together, to recruit them for the Register, to supply them with ample knowledge, and to fire them with zeal for the best interests of their profession and themselves. We hope that steps may be taken without further delay to move the women members of the Council to organise and equip themselves for the active crusade which lies before them in the near future, if victory is to be promptly won for the College, the nursing profession, and the cause in which they are interested.

Will not the more influential and ready of the matron members of the College Council take an opportunity to assemble their sister members and to set about organising every member of that Council to utilise to the full the special influence in particular fields which each member can bring to bear to help forward the successful issue of the great scheme of which Mr. Stanley has been elected President? The sooner stock is taken of the resources and forces of the College and its supporters in regard to propaganda and the speedy raising of the number of nurses on the Register to overwhelming numbers, the sooner will Nurses' Registration be recognised by statute and placed in the hands of those who, by training and knowledge, have the best claim to take an active part in the administration of the affairs of the College and Register.

It will be helpful to all engaged in the forthcoming crusade on behalf of the nursing profession to remember that the present position of nursing affairs has produced three parties: one which is opposed to the College and to registration in any form or shape; two, the overwhelming majority of trained nurses who are sincerely anxious to have a British College of Nurses under the best auspices, and to secure the registration of nurses on the highest possible basis in the shortest possible time; and thirdly, the section of one woman, who wishes to keep everything in her own hands and to hold that position by denouncing much that is self-respecting and worthy in the nursing world. Irreconcilables and the sowers of disunion have never prospered in the world at large. They are not likely to attain any real vitality in the world of nursing in Great Britain at the present time. Still there are these three sections or parties, and the smallness and relative unimportance, having regard to the whole field of nursing, of the first and last of them should not blind the College and registration party to the necessity for them to be up and doing. Untiringly and actively working for the development of Mr. Stanley's remarkable scheme and the furtherance of its success by continuous, wisely directed effort until the goal is reached.

That goal is an amply endowed College of Nursing conceived and organised upon the highest possible standards, in combination with a system of nurse registration which will protect the nurse and the public whilst it raises the standard of nurse education, efficiency, and character. Mr. Stanley has been doing and is doing yeoman service by attending meetings up and down the country and meeting objectors and anxious inquirers. Miss Gill and the Association for the Promotion of Registration of Nurses in Scotland have deservedly won the sympathy and support of the overwhelming majority of nursing institutions, hospitals, and nurses in Scotland for the College of Nursing and State Registration. We should like to see the greater English and Welsh matrons up and actively at work in the same direction, for they can quite readily, if they will, with the aid of the sisters and the nurses they have trained, win equal support for Mr. Stanley's proposals throughout England and Wales. Will they not bestir themselves and pursue an untiring, wisely conceived, and ably conducted propaganda throughout the country?

Mrs. Bedford Fenwick has overstrained her bow. We are in a position to know and to state, what is well understood by matrons of importance throughout the United Kingdom, that her sisters in the nursing world have extended to her the utmost consideration and forbearance, and that they were entitled to expect a better result than that she should suddenly turn round and devote her great energy to the endeavour to sow disunion. That she cannot succeed is abundantly testified by the powerful protests which she has excited. They embody strong remonstrances against her unconstitutional and uncalled for policy. It is not necessary for us to add anything to these protests. We content ourselves with publishing in full in another column that of the Association for the Promotion of Registration of Nurses in Scotland.

